# nCounter^®^ PanCancer Immune Profiling Panel (NanoString Technologies, Inc., Seattle, WA)

**DOI:** 10.1186/s40425-015-0088-7

**Published:** 2015-12-15

**Authors:** Alessandra Cesano

**Affiliations:** NanoString Technologies, Inc., Seattle, WA USA

## ᅟ

**Fig. 1:**
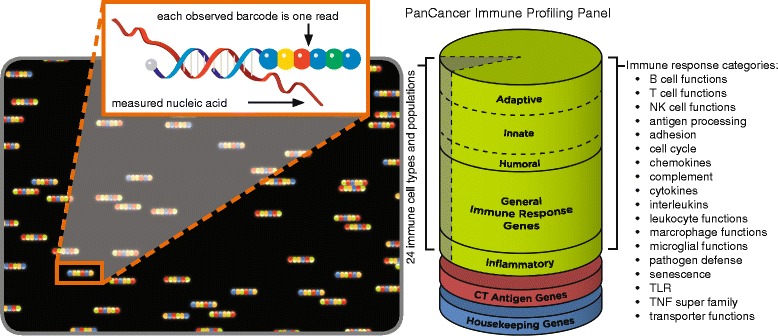
(*left*) Molecular barcoding. NanoString Molecular barcodes consist of 6 fluorescent “spots” that can be any one of 4 colors each [hence arranged in 4^6 combinations (4096), of which 800 have been commercialized], each specific color combination represents a single mRNA species to be measured. The mRNA from the sample of interest (red) hybridizes to the complementary nucleic-acid attached to an optical barcode pattern representing that specific gene. While the hybridization event occurs in solution, the final digital counting of the barcode-nucleic-acid component is performed, single molecule at-a-time, stretched-out on the surface of the nCounter cartridge (spot-pattern on black background). (*right*) Schematic representation of the PanCancer Immune Profiling Panel. This 770-plex assay contains: 109 genes to cell surface markers capable of quantitating 24 different tumor infiltrating immune cell types and populations, 30 genes for commonly studied CT Antigens, over 500 genes for measuring immune response with a special emphasis on checkpoint regulation/signaling, 40 PanCancer Reference Genes. The panel can be further “customized” (by individual researchers) with the addition of up to 30 genes with our Panel-Plus feature.

The *nCounter PanCancer Immune Profiling Panel* is a unique 770-plex gene expression panel to measure the human immune response in both solid and liquid cancer types. The panel measures many features of the immune response to facilitate rapid development of clinical actionable gene expression profiles in the context of cancer immunotherapy. The assay is run on the *nCounter Analysis System* (Nanostring Technologies, Inc.), an automated system which received 510(k) clearance from the FDA for use with the Prosigna Breast Cancer Prognostic Gene Signature Assay [1]. The *nCounter Analysis System* is based on a novel digital color-coded barcode technology which allows for direct multiplexed measurement of gene expression from low amount of mRNA (25 to 300 ng) without need for amplification [2]. The technology uses molecular “barcodes” and single molecule imaging to detect and count (completely digital) hundreds of unique transcripts in a single reaction with high precision and sensitivity (<1 copy per cell). Each color-coded optical barcode is attached to a single target-specific hybridization probe corresponding to a gene of interest. Mixed together with controls, they form multiplexed “CodeSets” which are provided as ready to use reagents (Fig. 1). Currently three “CodeSets” are available as ready to use reagents for oncology investigations: a) the *PanCancer Pathways* - a panel of 700 essential genes representing major cancer pathways including key driver genes - b) the *PanCancer progression* - a panel of 770 genes from 4 major biologic processes that contribute to increased tumor growth and invasiveness including angiogenesis, epithelial to mesenchymal transition and extra-matrix remodeling and metastasis and c) the PanCancer Immune profiling panel (described in more details below). Multiple CodeSets can be run on the same samples thus proving integrated information about both tumor and host immune response.

At a very high level, the assay includes three main steps:

1. **Hybridization**: unique pairs of a “capture” and a “reporter” probe are provided for each gene of interest, allowing up to 800 genes to be multiplexed, and their mRNA transcript levels measured, in a single experiment, for each sample. The “reporter” probe carries the signal, and the “capture” probe allows the complex to be immobilized for data collection.
2. **Purification and immobilization**: after hybridization, samples are transferred to the nCounter Prep Station where excess probes are removed and probe/target complexes are bound, immobilized, and aligned on the *nCounter Cartridge*.
3. **Counting and Analysis**: sample cartridges are placed in the *nCounter Digital Analyzer* for data collection.

The time from sample lysates to data results is two days and because the process is highly automated the hands-on time (and therefore room for human errors) is limited (25 min per 12 samples). Measurements are performed using the commercially available nCounter Analysis Instrumentation at the site of sample collection or through working with any of the multiple Contract Research Organizations offering NanoString services.

For application of this technology to immune-oncology the *nCounter PanCancer Immune Profiling Panel* provides a highly multiplexed gene expression panel designed to quantitate 770 genes that fall into four functional categories (Fig. 1):

1. **Identifying 24 different infiltrating immune cell types**, such as those in a peripheral blood mononuclear cells (PBMC) population or infiltrating into a tumor.
2. **Assessing immunological function** and response to immunotherapy, such as immune checkpoint regulation.
3. **Identifying tumor-specific antigens**, such as cancer-testis (CT) antigens.
4. **Housekeeping genes** that facilitate sample-to-sample normalization.

### Type of data obtained/readout

Barcodes are counted and tabulated for each target by the *nCounter Digital Analyzer*. The data readouts are either the composite of the individual expression profile of cells within that population; or detailed, single-cell level expression, which may represent biologically relevant small percentage (5-10 %) of the entire population of the cells. The instrument analysis software automatically performs QC, normalization, data analysis and creates multi-page reports with the options of performing advanced analyses including pathway applications.

### Limitations of the approach

- The data are not spatially resolved, hence, it represents the average of a few 1000’s of cells
- The data are targeted discovery (as contrasted with pure discovery), and measure only the 770 genes predefined in the panel. It is possible to add 30 completely custom genes to the 770-plex panel (total of 800-plex)
- While it is possible to resolve alternate splice transcripts and expressed gene-fusions, the panel does not measure single-nucleotide polymorphisms (SNPs).

### Advantages of the approach

- Multiplex hundreds of gene targets in a single reaction
- High sensitivity (<1 copy per cell)
- Fully-automated system
- No enzymes or amplification required to perform assay, ideally suited for FFPE samples and cell lysates
- Multiplex 800 regions from as little as 25-300 ng of total RNA
- Completely digital detection (all quantitation is by direct single-molecule counting)
- Automated analysis software

### Types of samples needed and special issues pertaining to samples

The nCounter PanCancer Immune profiling panel is fully compatible with clinically relevant sample types such as fresh-frozen (FF) tissue, formalin-fixed paraffin-embedded (FFPE) tumor sections, isolated immune cell populations such as PBMC and cell lysates. For very low input samples (even down to single-cell work, such at CTCs), a multiplexed target enrichment amplification protocol is available.

### Level of evidence

There have been bout over 800 peer-reviewed publications using the *nCounter Analysis System*. The instrument, reagents and software have received 510(k) clearance from the FDA for use with the Prosigna Breast Cancer Prognostic Gene Signature Assay [1]. There is a growing body of literature available demonstrating the use of the *nCounter analysis system* using much of the same content as in the *nCounter PanCancer Immune Profiling Panel* in immuno-oncology setting [3].

## Abbreviations

CT: Cancer testis antigens
CTC: Circulating tumor cells
FF: Fresh-frozen
FFPE: Formalin-fixed paraffin-embedded
FDA: Food and Drug Administration
PBMC: Peripheral blood mononuclear cells
QC: Quality check
RNA: Ribonucleic acid
SNPs: Single-nucleotide polymorphisms
